# Comprehensive microbiome causal mediation analysis using MiMed on user-friendly web interfaces

**DOI:** 10.1093/biomethods/bpad023

**Published:** 2023-10-04

**Authors:** Hyojung Jang, Solha Park, Hyunwook Koh

**Affiliations:** Department of Applied Mathematics and Statistics, The State University of New York, Korea, Incheon, South Korea; Department of Applied Mathematics and Statistics, The State University of New York, Korea, Incheon, South Korea; Department of Applied Mathematics and Statistics, The State University of New York, Korea, Incheon, South Korea

**Keywords:** causal mediation analysis, microbiome data analysis, web cloud computing, causal inference, human microbiome

## Abstract

It is a central goal of human microbiome studies to see the roles of the microbiome as a mediator that transmits environmental, behavioral, or medical exposures to health or disease outcomes. Yet, mediation analysis is not used as much as it should be. One reason is because of the lack of carefully planned routines, compilers, and automated computing systems for microbiome mediation analysis (MiMed) to perform a series of data processing, diversity calculation, data normalization, downstream data analysis, and visualizations. Many researchers in various disciplines (e.g. clinicians, public health practitioners, and biologists) are not also familiar with related statistical methods and programming languages on command-line interfaces. Thus, in this article, we introduce a web cloud computing platform, named as MiMed, that enables comprehensive MiMed on user-friendly web interfaces. The main features of MiMed are as follows. First, MiMed can survey the microbiome in various spheres (i) as a whole microbial ecosystem using different ecological measures (e.g. alpha- and beta-diversity indices) or (ii) as individual microbial taxa (e.g. phyla, classes, orders, families, genera, and species) using different data normalization methods. Second, MiMed enables covariate-adjusted analysis to control for potential confounding factors (e.g. age and gender), which is essential to enhance the causality of the results, especially for observational studies. Third, MiMed enables a breadth of statistical inferences in both mediation effect estimation and significance testing. Fourth, MiMed provides flexible and easy-to-use data processing and analytic modules and creates nice graphical representations. Finally, MiMed employs ChatGPT to search for what has been known about the microbial taxa that are found significantly as mediators using artificial intelligence technologies. For demonstration purposes, we applied MiMed to the study on the mediating roles of oral microbiome in subgingival niches between e-cigarette smoking and gingival inflammation. MiMed is freely available on our web server (http://mimed.micloud.kr).

## Introduction

The human microbiome is the totality of all microbes that live on and inside various organs (e.g. gut, mouth, skin, and nose) of the human body. The advances in massively parallel metagenomic sequencing have dramatically lowered the cost of microbiome profiling with a substantial increase in accuracy. Then, the microbiome field has not only become an active area of research, but also rapidly grown in industry with the aim of identifying new ways to diagnose, treat, and prevent human diseases.

**Figure 1.**
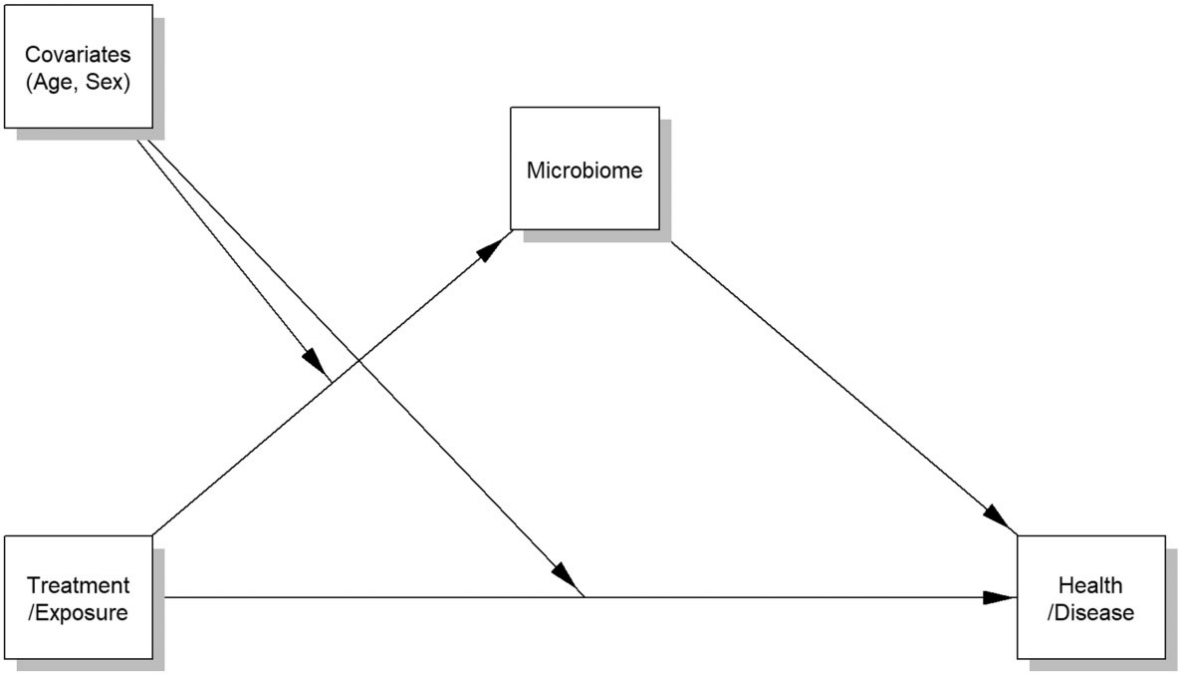
A conceptual illustration of the roles of the microbiome as a mediator between a treatment/exposure and a health or disease outcome with potential covariate effects.

Researchers have revealed a sophisticated interplay between microbiome and its host in various aspects. For instance, microbiome diversity and its taxonomic composition have been related to a variety of environmental, behavioral, or medical exposures (e.g. diet [1], residence [2], smoking [3], preterm birth [4], delivery mode [5, 6], and antibiotic/probiotic use [7, 8]). Researchers have also found that microbiome dysbiosis can lead to numerous disorders (e.g. obesity [9, 10], intestinal disease [11–13], cancers [14–16], diabetes [8, 17], and brain disorders [18, 19]). However, beyond such separate discoveries, it is essential to understand if the microbiome transmits the effects of environmental, behavioral, or medical exposures (say, treatment) to health or disease outcomes (say, outcome) as a mediator (Fig. 1), which can be surveyed through causal mediation analysis [20].

Mediation analysis aims to comprehend the underlying mechanism in an observed relationship between a treatment and an outcome through a third hypothetical variable, known as a mediator, indirectly. That is, in human microbiome studies, mediation analysis surveys two links jointly, (i) the effect of a treatment on microbiome (denoted as “treatment—microbiome”) and (ii) the effect of microbiome on an outcome conditional on treatment status (denoted as “microbiome—outcome”) (Fig. 1). If we lose any one of these two links, microbiome does not serve as a mediator. That is, if we have “treatment—microbiome” but do not have “microbiome—outcome,” the treatment alters microbiome, but the altered microbiome has no effect on the outcome. This means that the effect of the treatment on the outcome was made “directly” or by some other unknown pathways, not through the microbiome. Similarly, if we do not have “treatment—microbiome” but have “microbiome—outcome,” the treatment does not alter the microbiome, but only the variability in microbiome due to some other unknown sources influences the outcome. Thus, the roles of the microbiome as a mediator are satisfied only when we have both links [20], which we refer as the presence of “indirect” or “mediation” effect. It substantially matters in a clinical context because if the microbiome is not in a causal pathway, any medical interventions to the microbiome do not fundamentally treat or prevent human diseases.

However, in human microbiome studies, mediation analysis is not used as much as it should be. One reason is because of the lack of carefully planned routines, compilers, and automated computing systems [21] for microbiome mediation analysis (MiMed) to perform a series of data processing, diversity calculation, data normalization, downstream data analysis, and visualization. The microbiome data are highly complex, and also demand many data processing and analytic procedures. Many researchers in various disciplines (e.g. clinicians, public health practitioners, and biologists) are not also familiar with related statistical methods and programming languages on command-line interfaces. Moreover, there are many other important issues that need to be addressed for microbiome causal mediation analysis as follows. First, we can view the microbiome as a whole community in an ecological context (referred in this article for “community-level analysis”) or can focus on individual microbial taxa at various taxonomic hierarchies (i.e. phyla, classes, orders, families, genera, and species) (referred in this article for “taxonomy-level analysis”). Researchers usually survey the former using different ecological measures (e.g. alpha- and beta-diversity indices) [22, 23] and the latter using different data normalization methods (e.g. centered-log ratio (CLR) [24], arcsine-root, proportion). Second, covariate-adjusted analysis is needed to control for potential confounding factors (e.g. age and gender), which is especially necessary for observational studies to enhance the causality of the results. Third, both mediation effect estimation and significance testing are important portions of statistical inference for better interpretability. Fourth, we need flexible and easy-to-use data processing and analytic modules as well as high-quality visualizations, for example, to be included in an academic paper. Finally, we need to figure out what have been known about the microbes that we discovered as significant mediators. However, it is not straightforward in practice to figure it out all manually since there are too many microbial taxa [25] and related prior studies. Hence, we may need a well-trained artificial intelligence (AI) machine that can do such a job for us.

To tackle all the critical issues described above, here we introduce a web cloud computing platform, named as MiMed, that enables comprehensive MiMed on user-friendly web interfaces. MiMed is the first web cloud computing platform for microbiome causal mediation analysis, which is distinguished from our prior platforms: (i) MiCloud for association analysis in cross-section or longitudinal microbiome studies [26]; (ii) MiPair for design-based comparative analysis with paired microbiome data [27]; and (iii) MiSurv for microbiome data analysis with survival responses [28]. Interestingly, MiMed also builds-in access to the popular AI language model, ChatGPT, to easily search for what have been known about the microbial taxa that are found significantly as mediators. We note that this plug-in facility for ChatGPT is for quick and easy check-ups, and, of course, the results from ChatGPT are not always right. Thus, we would suggest using it with caution. For verification purposes, we also had MiMed report the search results from Google Scholar and PubMed along with the results from ChatGPT.

In the following “Materials and methods” section, we describe the methodological ideas of causal mediation analysis methods as well as our web server and local GitHub repository. Then, in the “Results” section, we describe all the data processing and analytic modules one by one using an example study to see the mediating roles of oral microbiome between e-cigarette smoking and gingival inflammation [29]. Finally, in the “Discussion” section, we summarize and discuss all the features and implications of MiMed. MiMed is freely available on our web server (http://mimed.micloud.kr) or can alternatively run on a user’s local computer (https://github.com/yj7599/MiMedGit).

## Materials and methods

### Statistical methods

This section is devoted to describing the methodological aspects of the causal mediation analysis methods. We describe only the conceptual ideas and terms to help our users to easily understand them, while referencing the original papers for all technical details.

To begin with the Sobel test [30], Preacher–Hayes approach [31, 32]) and Divide-Aggregate Composite-null Test (DACT) [33], the Baron and Kenny’s two regression models [20] below can first be considered.


(1) $$M_{i}=\;\alpha_{0}\;+\;\alpha_{1}T_{i}\;+\;\varepsilon_{i}$$

(2) $$Y_{i}\;=\;\beta_{0}\;+\;\beta_{1}M_{i}\;+\;\beta_{2}T_{i}\;+\;\upsilon_{i}$$

where $T_{i}$ is a treatment, $M_{i}$ is a mediator (e.g. an alpha-diversity index or a microbial taxon), $Y_{i}$ is a health or disease outcome, $\alpha_{0}$ and $\beta_{0}$ are intercepts, $\alpha_{1}$, $\beta_{1},\;$and $\beta_{2}$ are slopes, and $\varepsilon_{i}$ and $\upsilon_{i}$ are independently distributed random errors for the units *i *=* *1, …, *n*. To ease our demonstration, we suppose in addition that $T_{i}$ is a binary treatment variable ($T_{i}$* *=* *0 for control and $T_{i}$* *=* *1 for treatment) and $Y_{i}$ is a continuous health or disease outcome variable. Yet, more extensions are available (Table 1). Then, the null and alternative hypotheses below are considered


(3) $$H_{0}:\;\alpha_{1}\beta_{1}\;=\;0\;\mathrm{vs}.\;\;H_{1}:\;\alpha_{1}\beta_{1}\;\neq \;0.$$

**Table 1.** Descriptive table for the functionalities of causal mediation analysis methods: Imai method, Sobel test, Preacher–Hayes approach, DACT, and MedTest.

			Community-level analysis					Taxonomy-level analysis		
			Alpha diversity				Beta diversity			
Treatment variable	Outcome variable		Imai (Default)	Sobel	Preacher–Hayes	DACT	MedTest (Default)	Imai (Default)	Sobel	DACT
Binary	Binary	Interaction	O	X	X	X	X	O	X	X
		Covariates	O	X	O	O	O	O	X	O
		Point estimation	O	X	O	O	X	O	X	O
		Interval estimation	O	X	O	X	X	O	X	X
		*P*-value	O	X	X	O	O	O	X	O
	Continuous	Interaction	O	X	X	X	X	O	X	X
		Covariates	O	X	O	O	O	O	X	O
		Point estimation	O	O	O	O	X	O	O	O
		Interval estimation	O	X	O	X	X	O	X	X
		*P*-value	O	O	X	O	O	O	O	O
Continuous	Binary	Interaction	O	X	X	X	X	O	X	X
		Covariates	O	X	O	O	O	O	X	O
		Point estimation	O	X	O	O	X	O	X	O
		Interval estimation	O	X	O	X	X	O	X	X
		*P*-value	O	X	X	O	O	O	X	O
	Continuous	Interaction	O	X	X	X	X	O	X	X
		Covariates	O	X	O	O	O	O	X	O
		Point estimation	O	O	O	O	X	O	O	O
		Interval estimation	O	X	O	X	X	O	X	X
		*P*-value	O	O	X	O	O	O	O	O

*Note*: “O” represents that the method can handle/address it, while “X” represents that the method cannot handle/address it.

Here, $\alpha_{1}$ represents the effect of the treatment ($T_{i}$) on the mediator ($M_{i}$) as in Equation (1) and $\beta_{1}$ represents the effects of the mediator ($M_{i}$) on the outcome ($Y_{i}$) conditional on treatment status ($T_{i}$) as in Equation (2). Then, the null hypothesis, $H_{0}$: $\alpha_{1}\beta_{1}$* *=* *0, states that at least one of $\alpha_{1}$ and $\beta_{1}$ equals to zero indicating no mediation effect, while the alternative hypothesis, $H_{1}$: $\alpha_{1}\beta_{1}$ ≠ 0, states that both $\alpha_{1}$ and $\beta_{1}$ are non-zero indicating the presence of mediation effect. The Sobel test [30] conducts significance testing for Equation (3) using a parametric approach that assumes that $\varepsilon_{i}$ and $\upsilon_{i}$ in Equations (1) and (2) are normally distributed. In contrast, the Preacher–Hayes approach [31, 32] does it non-parametrically using a bootstrap method [34] without the normality assumption. As for the Sobel test [30], DACT [35] is a parametric approach, but considers the null hypothesis, $H_{0}$: $\alpha_{1}\beta_{1}$* *=* *0, in Equation (3) as a composite hypothesis that $H_{0}$: (i) $\alpha_{1}$* *=* *0 and $\beta_{1}$ ≠ 0; (ii) $\alpha_{1}$ ≠ 0 and $\beta_{1}$* *=* *0; or (3)$\alpha_{1}$* *=* *0 and $\beta_{1}$* *=* *0; to improve statistical power while rejecting $H_{0}$ for at least one of the three sub-statements.

As for DACT [33], MedTest [35] considers the null hypothesis as a composite hypothesis, but it is a non-parametric significance test based on a permutation method. A more important distinction is that MedTest [35] formulates the mediator ($M_{i}$) in Equations (1) and (2) as a function of beta-diversity (say, ${f(M)}_{i}$, where f(.) is a function that transforms microbiome into a eta-diversity index); as such, it enables causal mediation analysis for beta-diversity (Table 1).

We can classify the Sobel test [30], Preacher–Hayes approach [31, 32], DACT [33], and MedTest [35] as “product-of-coefficients” methods because of their shared hypothesis of Equation (3) in the form of $\alpha_{1}\beta_{1}$ (i.e. the product of coefficients from Equations (1) and (2)). However, the Imai method [36, 37] in contrast is based on the potential outcomes framework of causal inference [38], i.e. $Y_{i}(T_{i}$, $M_{i}$($T_{i}$)), where the level of health or disease outcome is a function of a treatment status (i.e. $T_{i}$) and the level of the mediator under a treatment status (i.e. $M_{i}$($T_{i}$)). Then, the unit-level “total treatment effect” can be defined as Equation (4), the unit-level “direct effect (DE)” on the mediator can be defined for each treatment status (*t *=* *0 for control or *t *=* *1 for treatment) as Equation (5), and finally the unit-level “indirect effect or causal mediation effect (CME)” can be defined for each treatment status (*t *=* *0 for control or *t *=* *1 for treatment) as Equation (6),


(4) $$\tau_{i}\;\equiv \;Y_{i}(1,\;M_{i}(1))\;-\;Y_{i}(0,\;M_{i}(0)).$$

(5) $$\zeta_{i}(t)\;\equiv \;Y_{i}(1,\;M_{i}(t))\;-\;Y_{i}(0,\;M_{i}(t)).$$

(6) $$\delta_{i}(t)\;\equiv \;Y_{i}(t,\;M_{i}(1))\;-\;Y_{i}(t,\;M_{i}(0)).$$

Here, the unit-level total treatment effect in Equation (4) was formulated by subtracting the level of health or disease outcome for the unit under control and the level of the mediator under control from the level of health or disease outcome for the same unit under treatment and the level of the mediator under treatment. The unit-level DE for each treatment status (i.e. for control or treatment) in Equation (5) was formulated by subtracting the level of health or disease outcome for the unit with under control from the level of health or disease outcome for the same unit under treatment. Finally, the unit-level CME for each treatment status (i.e. for control or treatment) in Equation (6) was formulated by subtracting the level of health or disease outcome for the unit with the level of the mediator under control from the level of health or disease outcome for the same unit with the level of the mediator under treatment.

Then, the overall “average direct effect (ADE)” can be found by $\frac{1}{2}(\frac{1}{n}\sum_{i=1}^{n}\zeta_{i}\left(0\right)+\frac{1}{n}\sum_{i=1}^{n}\zeta_{i}\left(1\right))$, i.e. the average between the ADE with the level of mediator under control, $\frac{1}{n}\sum_{i=1}^{n}\zeta_{i}(0)$and the ADE with the level of mediator under treatment, $\frac{1}{n}\sum_{i=1}^{n}\zeta_{i}(1)$. Finally, the overall “average causal mediation effect (ACME),” i.e. the main result in causal mediation analysis, can be found by $\frac{1}{2}(\frac{1}{n}\sum_{i=1}^{n}\delta_{i}\left(0\right)+\frac{1}{n}\sum_{i=1}^{n}\delta_{i}\left(1\right))$ that is the average between the ACME for control, $\frac{1}{n}\sum_{i=1}^{n}\delta_{i}(0)$, and the ACME for treatment, $\frac{1}{n}\sum_{i=1}^{n}\delta_{i}(1)$. Especially, the Imai method [36, 37] also allows the interaction effect between the treatment ($T_{i}$) on the mediator ($M_{i}$) to be considered. For this, Imai *et al*. [37] extended Equations (2)–(7)


(7) $$Y_{i}\;=\;\gamma_{0}\;+\;\gamma_{1}T_{i}\;+\;\gamma_{2}M_{i}\;+\;\gamma_{3}T_{i}M_{i}\;+\;\varsigma_{i},$$

where $T_{i}M_{i}$ is the interaction term between $T_{i}$ and $M_{i}$, $\gamma_{0}$, $\gamma_{1}$, $\gamma_{2}$, and $\gamma_{3}$ are regression coefficients, and $\varsigma_{i}$ is an independently distributed random error for the units *i *=* *1, …, *n*. Then, based on Equations (1) and (7), Imai *et al.* [36, 37] showed that (i) the overall ADE can be found by $\frac{1}{2}[{\{\gamma }_{1}$ + $\gamma_{3}\alpha_{0}\}+\{\gamma_{1}$ + $\gamma_{3}(\alpha_{0}+\alpha_{1})\}]$, i.e. the average between the ADE with the level of mediator under control, $\gamma_{1}$ + $\gamma_{3}\alpha_{0}$, and the ADE with the level of mediator under treatment, $\gamma_{1}$ + $\gamma_{3}(\alpha_{0}+\alpha_{1})$, and (ii) the overall ACME can be found by $\frac{1}{2}[\{\alpha_{1}\gamma_{2}\}+\{\alpha_{1}(\gamma_{2}+\gamma_{3})\}]$, i.e. the average between the ACME for control, $\alpha_{1}\gamma_{2}$, and the ACME for treatment, $\alpha_{1}(\gamma_{2}+\gamma_{3})$. More details can be found in their original papers [36, 37].

The Imai method [36, 37] conducts interval estimation for ACME (overall) [as well as ACME (control), ACME (treatment), ADE (overall), ADE (control), ADE (treatment)] using a bootstrap method [34] non-parametrically, and its significance testing follows accordingly.

There has been a long debate on parametric versus non-parametric, but it is also beyond the scope of this article to make any resolute judgment on it. However, it is usual that non-parametric approaches are more robust to highly skewed data (e.g. rare taxa with excessive zeros), while parametric approaches are well suited to less skewed data (e.g. alpha-diversity indices or common taxa). However, so long as the sample size is large, the skewness does not also substantially matter for parametric approaches. However, it does not also mean that non-parametric approaches are not suited to a large sample size. Parametric approaches are not well suited to high skewed data with a small sample size. Since the microbiome data are usually highly skewed, we set non-parametric approaches as default, but we do not discourage the use of parametric approaches, which are also widely used and reasonable approaches for a large sample size (Table 1).

Of course, many other mediation analysis methods also exist. Especially for human microbiome studies, CMM [39, 40], SparseMCMM [41], microHIMA [42], LDM-med [43], and PERMANOVA-med [44] have recently been proposed. These methods might be promising to address the compositionality, high-dimensionality, sparsity, and/or phylogenetic structure of the microbiome data, and we do not depreciate them in methodological aspects. However, we could not incorporate them into MiMed because their software packages are not currently reliable (e.g. producing errors often) and/or their results are not easily interpreted with no parameter estimation or visualization facilities. We also believed that they need to gain more practical attention and be more widely used in the microbiome field to be available on web interfaces.

### Web server and local GitHub repository

We wrote all the user interfaces and server functions using R shiny (https://shiny.rstudio.com). We then developed our web server using ShinyProxy (https://www.shinyproxy.io) and Apache2 (https://httpd.apache.org) on the operating system, Ubuntu 20.04 (https://ubuntu.com). The web server currently runs on a computer with the specifications of Intel Core i9-12900 (16-core) processor and 64 GB DDR4 memory, and takes up to ten concurrent users. In case that the web server is busy, we also developed a local GitHub repository to enable to run MiMed using a user’s local computers. As usual, we, as a host, are responsible for and devoted to maintaining our web server and local GitHub repository reliable.

## Results

### Application note: on the roles of oral microbiome between e-cigarette smoking and gingival inflammation

To ease our demonstration, we use example data to survey the mediating roles of oral microbiome between e-cigarette smoking and gingival inflammation [29]. We refer to the original study paper [29] for all the details on study subjects, sample collection/processing, and sequencing/quantification procedures. To describe the portion of the data we use, the data are 16S oral microbiome data in subgingival niches obtained at the baseline visit of the subjects aged between 18 and 34 years. We employed a bioinformatic pipeline, QIIME2 [45], based on the expanded human oral microbiome database (eHOMD) [46] for raw sequence data processing, denoising, feature extraction/quantification, taxonomic annotation, and phylogenetic tree construction. We added detailed description on the use of each module using these example data at the end of each following section (see the “Application note” section).

### Data processing: data input

Microbiome data can be composed of three data components: (i) a feature table (i.e. count data for operational taxonomic units (OTUs) or amplicon sequence variants (ASVs)), (ii) a taxonomic table (i.e. taxonomic annotations at various taxonomic hierarchies, kingdom, phylum, class, order, family, genus, and species), and (iii) a phylogenetic tree (i.e. a rooted phylogenetic tree for evolutionary relationships across features, that are OTUs or ASVs). Of course, in addition to microbiome data, metadata on a treatment variable (e.g. environmental, behavioral, or medical exposures), an outcome variable (e.g. health or disease status), and possibly covariates (e.g. age and gender) for study subjects are needed. If we have all these data components, we can conduct microbiome causal mediation analysis comprehensively using all available functions of MiMed. However, researchers do not always have all these data components, but even in such a case, they can still want to conduct at least some parts of the analysis. Thus, we made the Data Input module flexible as the taxonomic table and/or the phylogenetic tree can be omitted. If the taxonomic table is omitted, only the community-level (alpha- and beta-diversity) analyses can be performed. If the phylogenetic tree is omitted, only the non-phylogenetic community-level (alpha- and beta-diversity) analyses can be performed.

Users can upload their data components in a widely used unified format, called phyloseq [47], or as separate files.

#### Application note

The example data we use can be downloaded in the Example Data section on the Data Input module. To help users to easily understand data components and their corresponding data analytic modules as described above, we uploaded four different sets of data components: (i) a feature table, a taxonomic table, a phylogenetic tree, and metadata; (ii) a feature table, a taxonomic table, and metadata; (iii) a feature table, a phylogenetic tree, and metadata; and (iv) a feature table and metadata. Since we aim in this article to describe all available functions of MiMed, we uploaded the one with all data components (i.e. a feature table, a taxonomic table, a phylogenetic tree, and metadata).

### Data processing: quality control

MiMed performs quality controls (QCs) just as in MiCloud [26] and MiPair [27]. That is, users need to select (i) a kingdom of interest (default: Bacteria), (ii) a minimum library size (i.e. total read count) for the study subjects to be rescued (default: 3000), (iii) a minimum mean relative abundance (i.e. proportion) for the features (OTUs or ASVs) to be rescued (default: 0.002%), and (iv) erroneous taxonomic names in the taxonomic table to be removed.

MiMed displays the sample size, the number of features (OTUs or ASVs), the number of phyla, the number of classes, the number of orders, the number of families, the number of genera, and the number of species using summary boxes before and after QCs. MiMed also visualizes library sizes across study subjects as well as mean proportions across features using interactive histograms and box plots before and after QCs.

#### Application note

**Figure 2.**
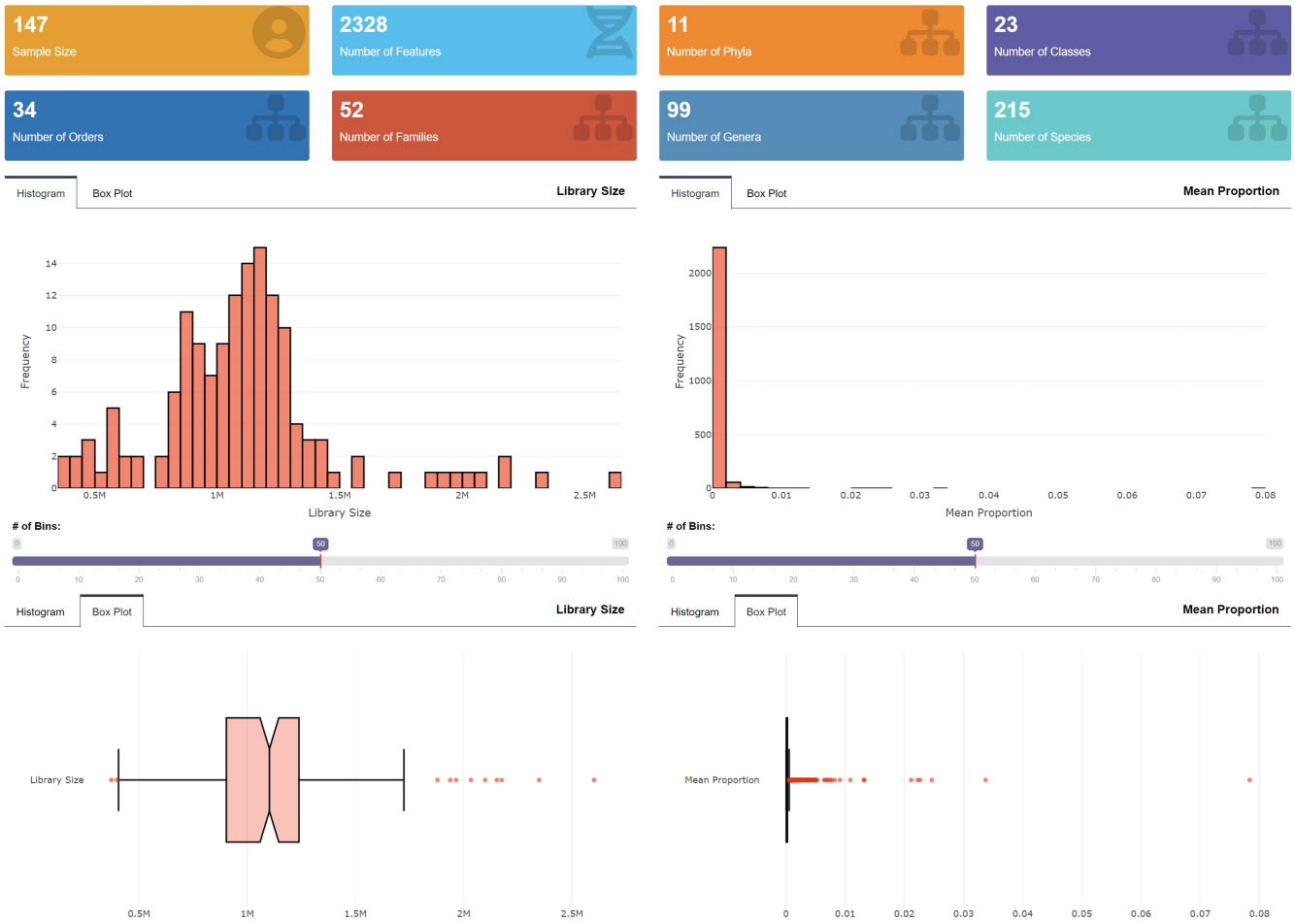
The status of the microbiome data after QCs. The summary boxes below display the sample size, the number of features, the number of phyla, the number of classes, the number of orders, the number of families, the number of genera, and the number of species after QCs. The histograms and box plots below visualize the library sizes across study subjects and the mean proportions across features.

We simply clicked the Run button to apply the default QC settings. Then, 147 subjects with 2328 features, 11 phyla, 23 classes, 34 orders, 52 families, 99 genera, and 215 species were retained in the following analyses (Fig. 2).

### Community-level analysis: diversity calculation

As in MiCloud [26], MiPair [27], and MiSurv [28], MiMed calculates nine alpha-diversity indices [i.e. eight non-phylogenetic indices: Observed, Shannon [48], Simpson [49], Inverse Simpson [49], Fisher [50], Chao1 [51], abundance-based coverage estimator (ACE) [52], incidence-based coverage estimator (ICE) [53]; 1 phylogenetic index: phylogenetic diversity (PD) [54]] and five beta-diversity indices (i.e. two non-phylogenetic indices: Jaccard dissimilarity [55], Bray–Curtis dissimilarity [56]; three phylogenetic indices: Unweighted UniFrac distance [57], Generalized UniFrac distance [58], and Weighted UniFrac distance [59]). For reference, users can download all the calculated alpha- and beta-diversity indices.

The term, diversity, itself is conceptual. Many researchers have thought about it for a long time, and they have formulated it all differently considering richness, evenness, and/or phylogeny, and also modulating them in different ways [48–59]. They have had different views on diversity, but it is not like which point of view or index is right or wrong. Different diversity indices can lead to different results in downstream statistical analyses. For example, some diversity indices can make statistically significant results, while others are not significant. It would make it hard to interpret the results with consistency, but it is also natural that they do not make consensus. For such a situation, we suggest interpreting the results listing the significant indices after the expression “according to” or “with respect to” as we did in later alpha- and beta-diversity analyses.

#### Application note

We simply clicked the Run button to calculate all the alpha- and beta-diversity indices.

### Community-level analysis: alpha diversity

This module analyzes if a treatment alters alpha-diversity, and then the altered alpha-diversity, in turn, influences an outcome, where the alpha-diversity can be surveyed using each of the nine alpha-diversity indices. For this, users first need to select (i) a treatment variable (e.g. diet, residence, smoking, preterm birth, delivery mode, and antibiotic/probiotic use), (ii) an outcome variable (e.g. health or disease status), (iii) to include an interaction term between a treatment and a mediator (alpha-diversity) in the model or not, and (iv) covariates (e.g. age and gender) to be adjusted for. We set the interaction term to be included (yes) as default since it is more natural to assume that the effect of microbiome on an outcome can be modulated by a treatment. That is, in order words, the effect of microbiome on an outcome can be different by treatment status. Ignoring the presence of such interaction effects may cause potential bias in mediation analysis [60, 61]. The only available analytic method that can address interaction effect is the Imai method [36, 37] (Table 1). The Imai method [36, 37] in addition allows covariate adjustments, estimates mediation effects in both point and interval estimation, and reports a *P*-value for significance testing. The other available analytic methods are two traditional (but still in wide use) methods, the Sobel test [30] and Preacher–Hayes approach [31, 32], and one recent method, DACT [33]. MiMed applies the Benjamini–Hochberg (BH) procedures [62]. MiMed visualizes the results from its alpha-diversity analysis using forest plots.

#### Application note

**Figure 3.**
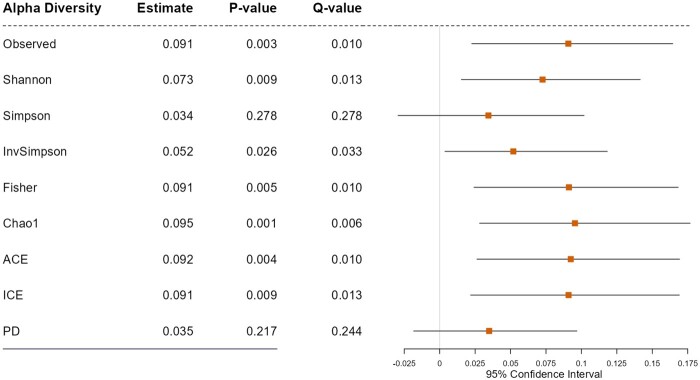
The results for alpha-diversity. We surveyed if e-cigarette smoking alters alpha-diversity of the oral microbiome in subgingival niches, and the altered alpha-diversity, in turn, influences gingival inflammation, adjusting for age, sex, and the frequency of brushing teeth. “Estimate” represents the ACME estimate.

We selected e-cigarette smoking as a treatment variable, gingival inflammation as an outcome variable, and age, sex, and the frequency of brushing teeth as covariates to be adjusted for in the presence of interaction between e-cigarette smoking and alpha-diversity. Then, we found significant results using the Imai method [36, 37] as e-cigarette smoking alters alpha-diversity of the oral microbiome in subgingival niches, and the altered alpha-diversity, in turn, influences gingival inflammation according to Observed, Shannon [48], InvSimpson [49], Fisher [50], Chao1 [51], ACE [52], and ICE [53] indices (Fig. 3).

### Community-level analysis: beta diversity

This module analyzes if a treatment alters beta-diversity, and then the altered beta-diversity, in turn, influences an outcome, where the beta-diversity can be surveyed using each of the five beta-diversity indices. For this, users first need to select (i) a treatment variable (e.g. diet, residence, smoking, preterm birth, delivery mode, and antibiotic/probiotic use), (ii) an outcome variable (e.g. health or disease status), and (iii) covariates (e.g. age and gender) to be adjusted for. MedTest [35] is currently the only available analytic method that can conduct causal mediation analysis for beta-diversity (Table 1). While MedTest [35] allows covariate adjustments and reports a *P*-value for significance testing, it is purely a test for significance with no facilities for mediation effect estimation not allowing any interaction term to be included (Table 1). MiMed applies the BH procedures [62]. MiMed visualizes the results from its beta-diversity analysis using principal coordinate analysis plots [63].

#### Application note

**Figure 4.**
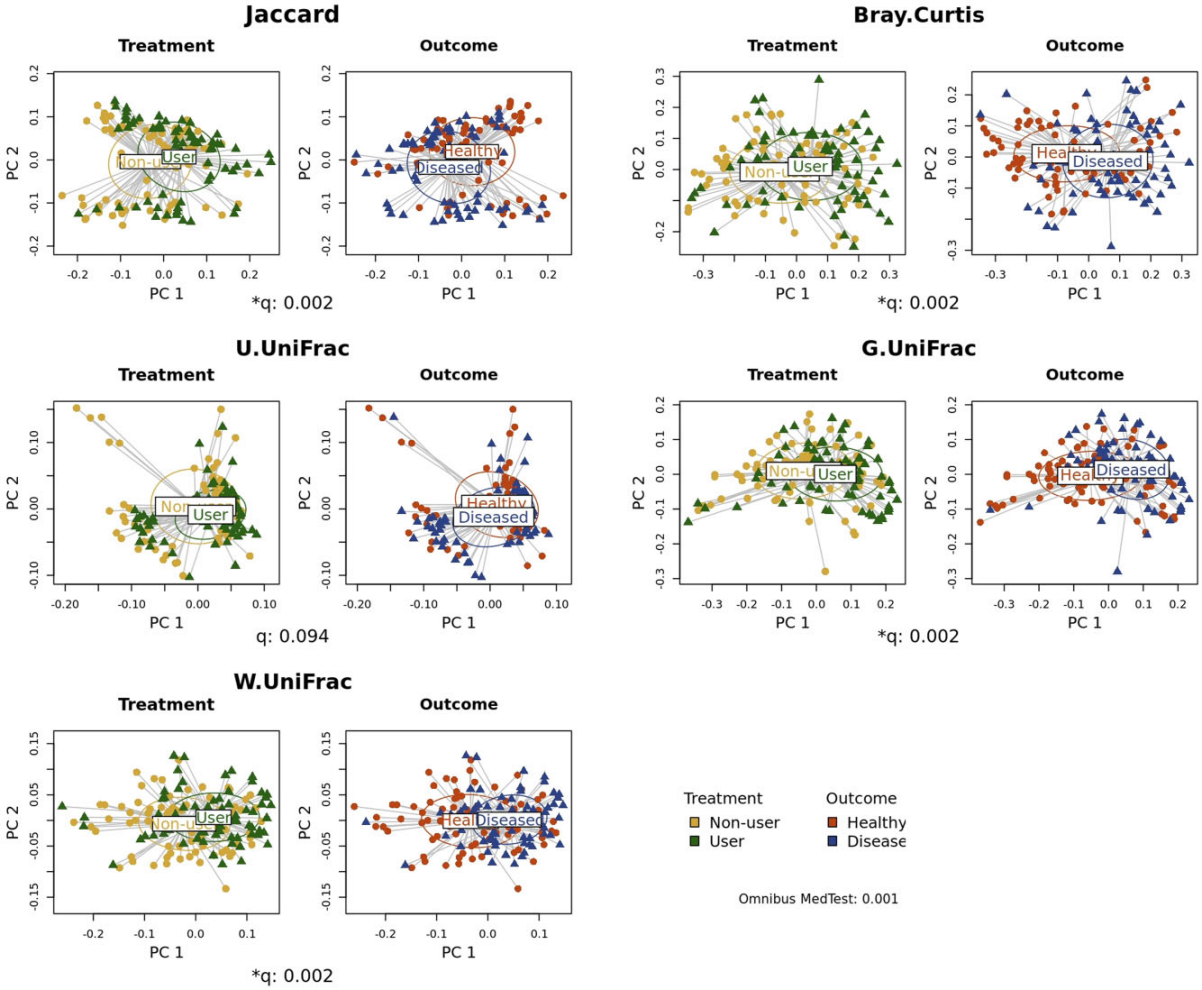
The results for beta-diversity. We surveyed if e-cigarette smoking alters beta-diversity of the oral microbiome in subgingival niches, and the altered beta-diversity, in turn, influences gingival inflammation, adjusting for age, sex, and the frequency of brushing teeth.

We selected e-cigarette smoking as a treatment variable, gingival inflammation as an outcome variable, and age, sex, and the frequency of brushing teeth as covariates to be adjusted for. Then, we found significant results using MedTest [35] as e-cigarette smoking alters beta-diversity of the oral microbiome in subgingival niches, and the altered beta-diversity, in turn, influences gingival inflammation according to Jaccard dissimilarity [55], Bray–Curtis dissimilarity [56], generalized UniFrac distance [58], and weighted UniFrac distance [59] (Fig. 4).

### Taxonomy-level analysis: data normalization

MiMed normalizes taxonomic absolute abundances (i.e. counts) through CLR [24], arcsine-root and proportion. The CLR transformation is the most widely used normalization method in the microbiome field to relax the compositional constraint while mapping the data in either absolute or relative abundance equivalently into real vector space [24]. The arcsine-root transformation is a traditional approach to stabilize the variance of relative abundances. That is, the variance of a binomial proportion close to 0.5 is larger than the one close to 0 or 1, but the arcsine-root transformation mitigates such a heteroscedasticity issue to be better suited to the conventional regression models under the assumption of homoscedasticity. The arcsine-root transformation has also recently been often used in the microbiome field [64]. Finally, the proportion is simply the relative abundance that can range from 0 to 1 to control for varying library sizes (i.e. total read counts) across study subjects. The proportion has the issues of compositional constraint and heteroscedasticity, but it is more intuitively recognized and interpreted than the data using CLR [24] or arcsine-root transformation. We set CLR [24] as default, and arcsine-root and proportion as user options in later taxonomic analysis based on their popularities. However, as we described above, both advantages and limitations exist for each of them, and thus it is beyond the scope of this article to make any resolute judgment on which data normalization method is the best.

For reference, users can download all the original count, proportion, and CLR and arcsine-root transformed taxonomic data for microbial taxa at various taxonomic hierarchies (i.e. phyla, classes, orders, families, genera, and species).

#### Application note

We simply clicked the Run button to normalize taxonomic relative abundances.

### Taxonomy-level analysis: taxonomic analysis

This module analyzes if a treatment alters microbial taxa, and then the altered microbial taxa, in turn, influence an outcome. For this, users first need to select a data format: CLR (default) [24], arcsine-root, or proportion. Users then need to select (i) a treatment variable (e.g. diet, residence, smoking, preterm birth, delivery mode, antibiotic/probiotic use), (ii) an outcome variable (e.g. health or disease status), (iii) to include an interaction term between a treatment and a mediator (taxon) in the model or not, and (iv) covariates (e.g. age and gender) to be adjusted for. Again, the only available analytic method that can address interaction effect is the Imai method [36, 37] (Table 1). Importantly, the Imai method [36, 37] is a non-parametric method based on a bootstrap approach [34]. Thus, it is highly robust against the high skewness of microbiome data, especially the rare microbial taxa with excessive zeros [36, 37]. The other available analytic methods are two parametric methods, the Sobel test [30] and DACT [33] (Table 1). We set the Imai method [36, 37] as default and the Sobel test [30] and DACT [33] as user options (Table 1), which is because of the robust performance of the Imai method [36, 37] as well as its broad range of functionalities (Table 1). To control for false discovery rates, MiMed applies the BH procedures [62] to each taxonomic hierarchy. MiMed visualizes the results from its taxonomic analyses using forest plots and dendrograms.

#### Ask ChatGPT

In this sub-module, users can ask ChatGPT a question: What is known about (discovered taxon) on (treatment) and (outcome)? For this, users first need to insert a ChatGPT API key that can be freely obtained on the website (https://platform.openai.com/account/api-keys). Then, users need to select a taxonomic rank (i.e. phylum, class, order, family, genus, and species) and a taxon that is discovered as a significant mediator. Then, users can rename the treatment and outcome variables using a human language replacing the original variable names that are hard to be recognized by ChatGPT. Then, ChatGPT will answer your question. However, ChatGPT is not always right. Especially, it is well-known that ChatGPT often provides fake references [65]. Thus, we added the search results from Google Scholar and PubMed for verification purposes at the bottom of the Ask ChatGPT module.

#### Application note

**Figure 5.**
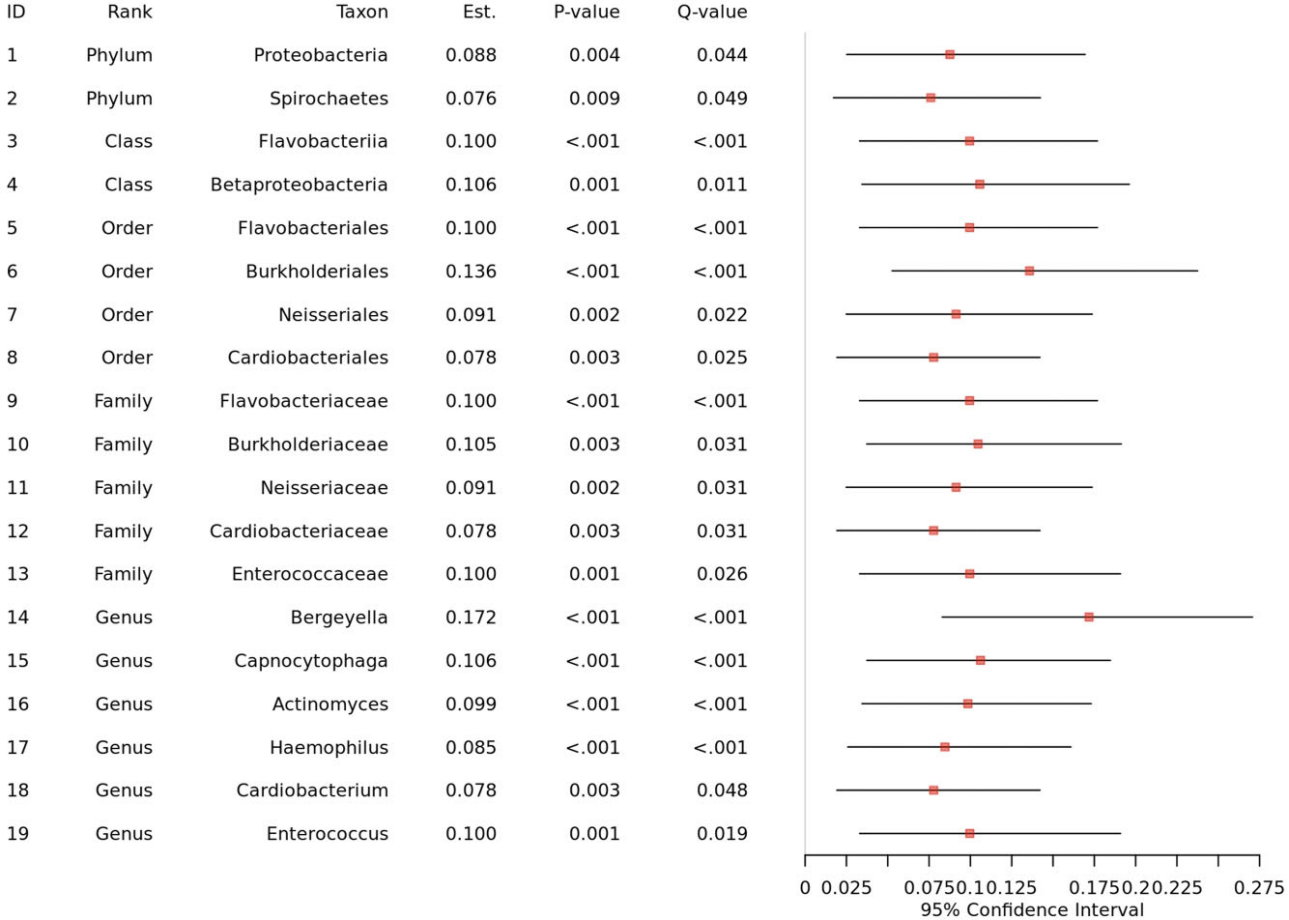
The results for microbial taxa. We surveyed if e-cigarette smoking alters the microbial taxa of the oral microbiome in subgingival niches, and the altered microbial taxa, in turn, influence gingival inflammation, adjusting for age, sex, and the frequency of brushing teeth.

**Figure 6.**
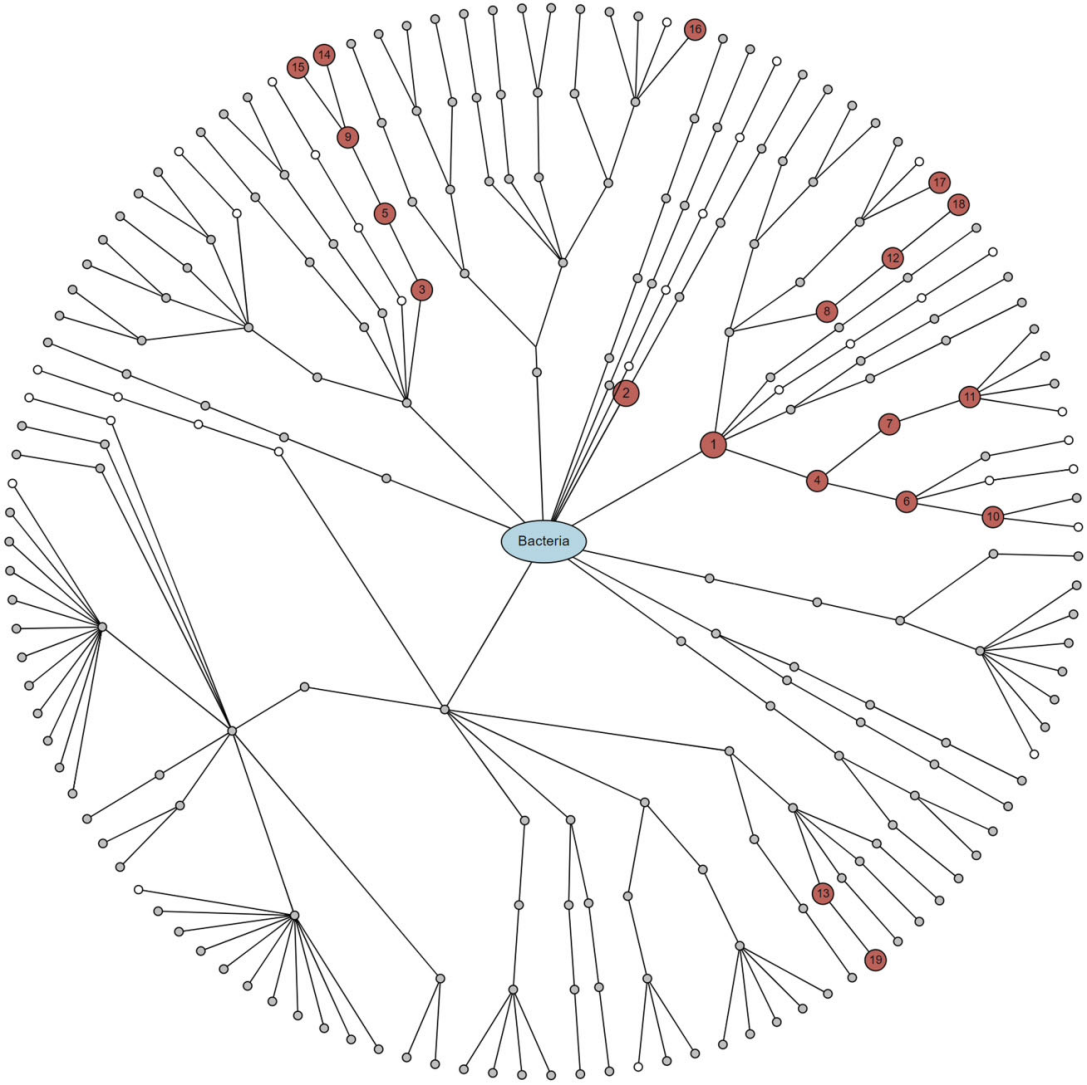
A hierarchical visualization for the taxonomic discoveries. The numbers in circles are matched with the IDs in Fig. 5. Red circle represents significant taxa, gray circle represents non-significant taxa, white circle represents the taxa that are not available in the taxonomic table to be tested.

**Figure 7.**
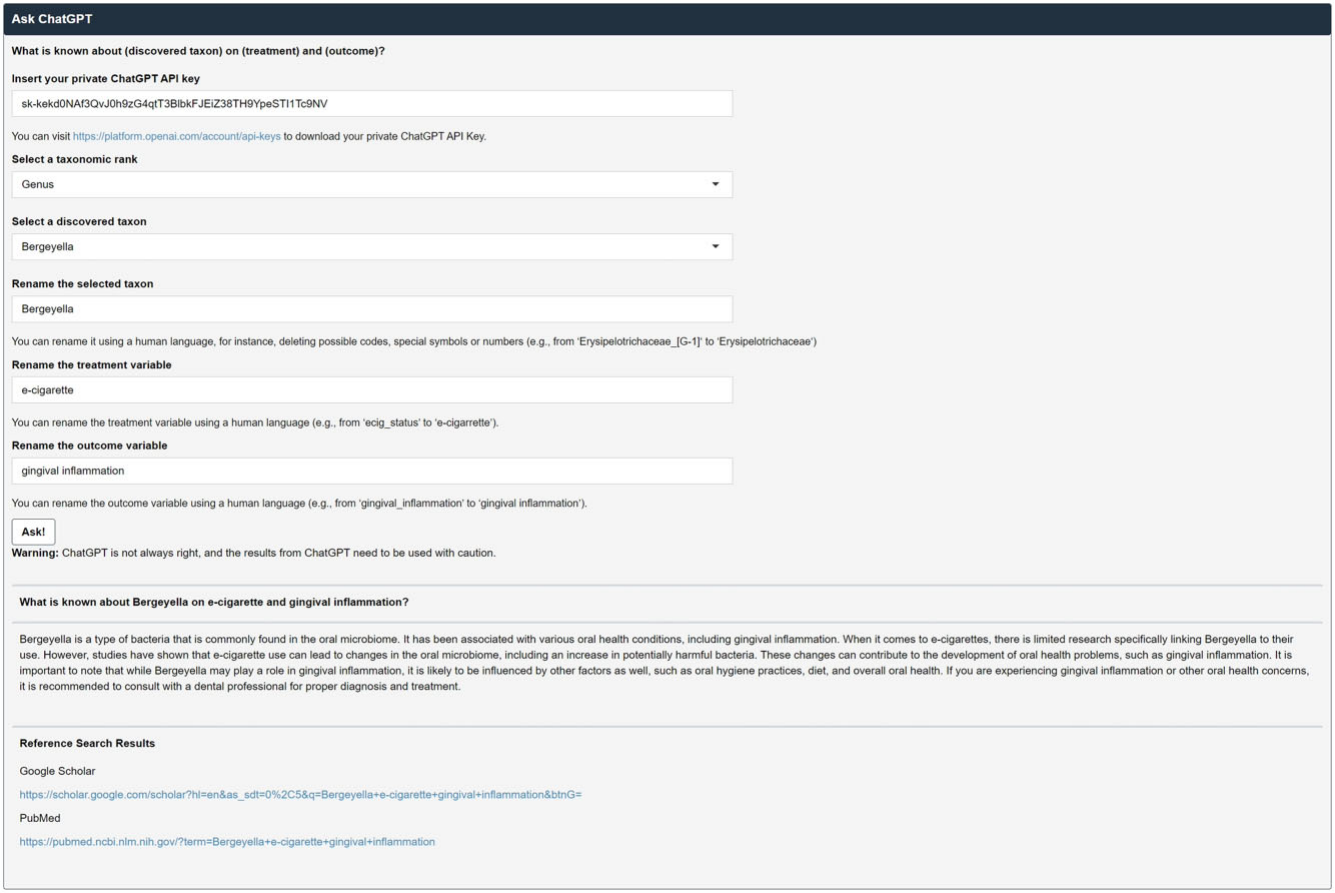
The screenshot of the Ask ChatGPT module. We asked ChatGPT a question, “What is known about *Bergeyella* on e-cigarette and gingival inflammation?” Then, ChatGPT answered the question. The Ask ChatGPT module also reports the search results from Google Scholar and PubMed at the bottom.

We selected CLR as a normalization method, e-cigarette smoking as a treatment variable, gingival inflammation as an outcome variable, and age, sex, and the frequency of brushing teeth as covariates to be adjusted for. Then, we found 21 significant taxa at the taxonomic hierarchies from phylum to genus (i.e. two phyla: Proteobacteria and Spirochaetes, two classes: Flavobacteriia and Betaproteobacteria, four orders: Flavobacteriales, Burkholderiales, Neisseriales, and Cardiobacteriales, five families: *Flavobacteriaceae*, *Burkholderiaceae*, *Neisseriaceae*, *Cardiobacteriaceae*, and *Enterococcaceae*, and eight genera: *Bergeyella*, *Capnocytophaga*, *Actinomyces*, *Haemophilus*, *Kingella*, *Burkholderia*, *Cardiobacterium*, and *Enterococcus*) using the Imai method [36, 37] as e-cigarette smoking alters their relative abundances, and the altered relative abundances, in turn, influence gingival inflammation (Figs 5 and 6). We also asked ChatGPT a question, “What is known about *Bergeyella* on e-cigarette and gingival inflammation?,” selecting a taxonomic rank as genus and a discovered taxon as *Bergeyella*, and renaming the treatment and outcome variables as e-cigarette and gingival inflammation. Then, ChatGPT aided to reconfirm our results answering as “*Bergeyella* is a type of bacteria that is commonly found in the oral microbiome. It has been associated with various oral health conditions, including gingival inflammation. When it comes to e-cigarettes, there is limited research specifically linking *Bergeyella* to their use. However, studies have shown that e-cigarette use can lead to changes in the oral microbiome, including an increase in potentially harmful bacteria. These changes can contribute to the development of oral health problems, such as gingival inflammation. It is important to note that while *Bergeyella* may play a role in gingival inflammation, it is likely to be influenced by other factors as well, such as oral hygiene practices, diet, and overall oral health. If you are experiencing gingival inflammation or other oral health concerns, it is recommended to consult with a dental professional for proper diagnosis and treatment.” (Fig. 7).

## Discussion

Researchers are interested in discovering causal mechanisms through which environmental, behavioral, or medical exposures influence health or disease outcomes. A promising approach has been to use mediation analysis, though it is highly demanding in the human microbiome field. The microbiome data are huge and highly complex, and many researchers are not familiar with dealing with such microbiome data. Thus, we need a well-designed “software” that enables user-friendly operations for microbiome causal mediation analysis.

In this article, we introduced MiMed, i.e. the first web cloud computing platform for microbiome causal mediation analysis. MiMed enables a long sequence of data processing and analytic operations on user-friendly web interfaces with widely extended flexibility and functionality. MiMed surveys the microbiome in various spheres as a whole ecosystem or as individual microbial taxa at various taxonomic hierarchies. MiMed also enables covariate-adjusted analysis and a breadth of statistical inferences in both mediation effect estimation and significance testing. MiMed also provides step-by-step data processing and analytic modules, and creates high-quality visualizations. Interestingly, MiMed also builds-in access to the recent popular chatbot, ChatGPT, to easily search for prior knowledge on discovered taxa using AI technologies. The plug-in facility for ChatGPT is helpful for quick and easy check-ups, but ChatGPT is not always right. Thus, we suggested using it with caution. Especially, for the fake reference issues [65], we added the search results from Google Scholar and PubMed for re-verification purposes.

MiMed is comprehensive and built with many data processing and analytic approaches. It is usual in the human microbiome field that there is no consensus on which approach is always the best. That is, there is not anything that is superior to the others in all contexts and situations. We are also curious about many different approaches. Thus, we left much room for our users to freely explore through many user options, while making a series of recommendations, as a developer, through default settings. For user’s convenience, MiMed also displays a list of references for the approaches that they use.

The human microbiome field is rapidly emerging and the microbiome data are recently flooded. Yet, the microbiome data are demanding and we are all so busy. Thus, MiMed can be attractive and useful in practice because it is user-friendly. MiMed will also provide new insights to the human microbiome field through causal mediation analysis that is too important to abandon [36].

## Data Availability

We used public microbiome data, where the raw sequence data are deposited at the NCBI Gene Expression Omnibus (http://www.ncbi.nlm.nih.gov/geo) under access number GSE201949. The processed data can also be found in the Example Data section on the Data Input module of MiMed (http://mimed.micloud.kr). MiMed is freely available on our web server (http://mimed.micloud.kr) or can alternatively run on a user’s local computer (https://github.com/yj7599/MiMedGit).
